# Sociodemographic considerations regarding the European Portuguese Validation of the Diabetes Distress Scale (DDS-17)

**DOI:** 10.20945/2359-4292-2026-0098

**Published:** 2026-08-23

**Authors:** Rodrigo Jesus Ccoto-Apolinario

**Affiliations:** 1 Instituto de Investigaciones en Ciencias Biomédicas, Universidad Ricardo Palma, Lima, Perú

Dear Editor,

The recent original article by Melo and cols. (^1^) successfully validated the Diabetes Distress Scale (DDS-17) for its implementation in the European Portuguese population. The introduction of this tool is of high clinical relevance, given that diabetes distress constitutes an affective dimension distinct from major depression and exerts a direct influence on therapeutic adherence and glycemic control. However, we consider that the sociodemographic characteristics of the evaluated cohort impose critical limitations that demand a cautious interpretation of the reported low levels of global distress.

In their study cohort, the mean age was 69.55 ± 12.55 years, and more than half of the participants (53.0%) had four years or less of education. On one hand, Almeida and cols. (^2^) demonstrated that perceived stress decreases in older adulthood due to reduced exposure to occupational stressors and lower reactivity to daily life conflicts. On the other hand, a low educational level is a well-known biasing factor in the use of self-report instruments. Data from the *All of Us* study (^3^) evidence that individuals with less than a high school education present an 11.6% rate of non-response or misunderstanding in stress scales, in contrast to the 5.3% observed in college graduates. Consequently, the reported low scores might not reflect a genuine absence of the condition, but rather marked differences in reading comprehension or cognitive fatigue within the sample.

Therefore, it would be pertinent to ask Melo and cols. whether a subgroup analysis regarding age and educational level was performed to clarify the variability of distress obtained in the study. In conclusion, we suggest that the clinical implementation of the DDS-17 in Portugal should rigorously account for these sociodemographic variables to mitigate the risk of false negatives. Furthermore, it would be advisable to complement questionnaire administration with strategies that ensure the correct interpretation of the items, such as assisted reading and a brief verification of question comprehension. Finally, we congratulate the authors for this valuable methodological contribution, which will undoubtedly optimize comprehensive disease management.

## STATEMENT OF USE OF GENERATIVE AI AND AI-ASSISTED TECHNOLOGIES IN THE WRITING PROCESS

During the process of preparation of this article, the author used Gemini (Google) for text structuring, stylistic refinement, and translation. After using this tool/service, the author reviewed and edited the content as necessary and assumes full responsibility for the content of the publication.

## Data Availability

datasets related to this article will be available upon request to the corresponding author.
